# Amoxicillin Increased Functional Pathway Genes and Beta-Lactam Resistance Genes by Pathogens Bloomed in Intestinal Microbiota Using a Simulator of the Human Intestinal Microbial Ecosystem

**DOI:** 10.3389/fmicb.2020.01213

**Published:** 2020-06-04

**Authors:** Lei Liu, Qing Wang, Huai Lin, Ranjit Das, Siyi Wang, Hongmei Qi, Jing Yang, Yingang Xue, Daqing Mao, Yi Luo

**Affiliations:** ^1^College of Environmental Science and Engineering, Ministry of Education Key Laboratory of Pollution Processes and Environmental Criteria, Nankai University, Tianjin, China; ^2^Hebei Key Laboratory of Air Pollution Cause and Impact (preparatory), College of Energy and Environmental Engineering, Hebei University of Engineering, Handan, China; ^3^Key Laboratory of Environmental Protection of Water Environment Biological Monitoring of Jiangsu Province, Changzhou Environmental Monitoring Center, Changzhou, China; ^4^School of Medicine, Nankai University, Tianjin, China

**Keywords:** amoxicillin, antibiotic resistance genes (ARGs), functional pathway genes, human intestinal microbiota, simulator of the human intestinal microbial ecosystem (SHIME)

## Abstract

Antibiotics are frequently used to treat bacterial infections; however, they affect not only the target pathogen but also commensal gut bacteria. They may cause the dysbiosis of human intestinal microbiota and consequent metabolic alterations, as well as the spreading of antibiotic resistant bacteria and antibiotic resistance genes (ARGs). *In vitro* experiments by simulator of the human intestinal microbial ecosystem (SHIME) can clarify the direct effects of antibiotics on different regions of the human intestinal microbiota, allowing complex human microbiota to be stably maintained in the absence of host cells. However, there are very few articles added the antibiotics into this *in vitro* model to observe the effects of antibiotics on the human intestinal microbiota. To date, no studies have focused on the correlations between the bloomed pathogens caused by amoxicillin (AMX) exposure and increased functional pathway genes as well as ARGs. This study investigated the influence of 600 mg day^–1^ AMX on human intestinal microbiota using SHIME. The impact of AMX on the composition and function of the human intestinal microbiota was revealed by 16S rRNA gene sequencing and high-throughput quantitative PCR. The results suggested that: (i) AMX treatment has tremendous influence on the overall taxonomic composition of the gut microbiota by increasing the relative abundance of *Klebsiella* [linear discriminant analysis (LDA) score = 5.26] and *Bacteroides uniformis* (LDA score = 4.75), as well as taxonomic diversity (Simpson, *P* = 0.067, *T*-test; Shannon, *P* = 0.061, *T*-test), and decreasing the members of *Parabacteroides* (LDA score = 4.18), *Bifidobacterium* (LDA score = 4.06), and *Phascolarctobacterium* (LDA score = 3.95); (ii) AMX exposure significantly enhanced the functional pathway genes and beta-lactam resistance genes, and the bloomed pathogens were strongly correlated with the metabolic and immune system diseases gene numbers (*R* = 0.98, *P* < 0.001) or *bl2_len* and *bl2be_shv2* abundance (*R* = 0.94, *P* < 0.001); (iii) the changes caused by AMX were “SHIME-compartment” different with more significant alteration in ascending colon, and the effects were permanent, which could not be restored after 2-week AMX discontinuance. Overall results demonstrated negative side-effects of AMX, which should be considered for AMX prescription.

## Introduction

Human intestinal microbiota co-exists in symbiosis with human beings and comprises with about 150 times more genes than the human genome, which makes intestinal microbiota become “another” genome of human beings (Qin et al., 2010). Moreover, human intestinal microbiota has been demonstrated to provide numerous important functions for the human health, including fermentation of indigestible dietary polysaccharides, synthesis of essential amino acids, and vitamins, modulation of the immune function and protection from the pathogens, as well as metabolism of the xenobiotic drugs (Chow et al., 2010; Yatsunenko et al., 2012; Cabreiro et al., 2013). Both human and veterinary antibiotics were detected in the collective gut of the Chinese population through our previous research (Wang et al., 2020). The antibiotics in our gut would kill or prevent the growth of commensal beneficial bacteria, which would cause the dysbiosis of intestinal microbiota, resulting multiple human diseases (Blaser, 2016; Ianiro et al., 2016; Flandroy et al., 2018; Kho and Lal, 2018). Antibiotics may also promote the spreading of antibiotic resistant bacteria (ARB) and antibiotic resistance genes (ARGs) in the human gut (Stecher et al., 2013; Barraud et al., 2018). One of the most commonly prescribed antibiotic in clinical and residential applications is amoxicillin (AMX), an inexpensive oral penicillin-type, beta-lactam antibiotic that kills a broad spectrum of bacteria by interfering with the synthesis of bacterial cell wall peptidoglycan layer (Zapata and Quagliarello, 2015; Zhang et al., 2015). The influence of AMX on human intestinal microbiota by *in vivo* research has been well studied and recorded, including human (Ladirat et al., 2014a; Pallav et al., 2014; Vrieze et al., 2014; Zaura et al., 2015; Oh et al., 2016) and human microbiota associated-animal models (Barc et al., 2008; Collignon et al., 2008; Collignon et al., 2010). Oh et al. (2016) noticed that AMX increased the abundance of *Klebsiella* in human intestinal microbiota. As a typical Gram-negative bacterial pathogen, *K. pneumoniae* is ubiquitous in the environment and symbioses in the human gut (Rock et al., 2014). However, the bloom of *K. pneumoniae* in human gut is often related to unhealthy status (Pena et al., 1997; Wiener-Well et al., 2010; Gorrie et al., 2017). Furthermore, no studies have suggested the bloom of *K. pneumoniae* caused by AMX exposure contributed to the increase of functional pathway genes and beta-lactam resistance genes.

Previous reports have revealed that changes of intestinal microbiota by AMX exposure are region-dependent and more significant effects were observed in the proximal colon than in the distal colon (Ladirat et al., 2014b; Marzorati et al., 2017). *In vivo* experiments detected the fecal samples that generally stand for the distal intestinal microbiota, which could not reveal the impacts of antibiotics on different gut regions. Moreover, *in vitro* experiment using simulator of the human intestinal microbial ecosystem (SHIME) can elucidate the direct effects of medicines on different regions of the human intestinal microbiota, which allows the complex human microbiota to be stably maintained in the absence of host cells (Van de Wiele et al., 2015). The SHIME model is known to be a useful tool for the *in vitro* study, which has already been used to identify the influence of bacteria and compounds on the colon microbiota, including probiotics such as *Clostridium cluster* XIVa and *Bifidobacterium longum* (Van den Abbeele et al., 2013; Truchado et al., 2015), prebiotics and prebiotics like compounds such as inulin, polyphenols and orange juice (Kemperman et al., 2013; Duque et al., 2016; Selak et al., 2016), and other toxic compounds such as Chlorpyrifos and Arsenic (Reygner et al., 2016; Yu et al., 2016). However, to the best of our knowledge, there are very few research that applied antibiotics into the SHIME model (Van den Abbeele et al., 2012; Bussche et al., 2015; Marzorati et al., 2017; Ichim et al., 2018; El Hage et al., 2019; Liu et al., 2020). These articles focused on the benefit of the mucosal environment, high-fiber diets, probiotic, and propionate-producing consortium in human intestinal microbiota. The effects of antibiotics, including AMX and other antibiotics mixture, vancomycin, and clindamycin, were limited on microbiota composition and metabolite. Also, there was little information available to demonstrate the link between the bloomed pathogens and functional pathway genes or ARGs. Hence, there is a need to study the influence of AMX on human intestinal microbiota in the SHIME model that integrates the entire gastrointestinal tract and maintains microbiome stability over an extended timeframe (Van de Wiele et al., 2015).

Since the reasonable dosage of AMX for the adult human study is about 750 to 1,500 mg day^–1^, and only half volume of the adult gut can be simulated in the SHIME model, here the direct effects of 600 mg day^–1^ of AMX on the composition and function of the human fecal microbiota were followed by the previous studies (Pallav et al., 2014; Reijnders et al., 2016). Three reactors (representing the ascending, transverse, and descending colon, respectively) that inoculated with human intestinal microbiota were studied for the three groups: immediately before AMX administration during 0 to 21 days (a control group), AMX-exposure during 22 to 28 days (an AMX treated group), and after the AMX discontinuance during 29 to 42 days (a recovery group). The 16S rRNA gene sequencing and high-throughput quantitative PCR (HT-qPCR) results revealed that AMX exposure caused a tremendous impact on the overall taxonomic composition of the gut microbiota, and increased functional pathway genes as well as ARGs. The changes were “SHIME-compartment” different with more significant modulation in the ascending colon, which could not be restored after 2-week AMX discontinuance. Therefore, the results of our research demonstrated a severe impact and a negative side-effect of AMX related to health problems, which should be considered as a fundamental aspect of the cost-benefit equation for its prescription.

## Materials and Methods

### The SHIME Model Experimental Setup and Sampling

The SHIME set up was formed by five double-jacketed reactors designated as the stomach, small intestine, ascending colon, transverse colon, and descending colon, respectively (Supplementary Figure S1). The last three reactors were inoculated with a mixture of fecal microbiota from a healthy adult volunteer, who did not suffer from gastrointestinal diseases or take antibiotics in the previous 6 months according to previous classic studies (Yu et al., 2016; Wang et al., 2018). And the differences between individuals may be alleviated by same culture condition (Van den Abbeele et al., 2013). The study was approved by the Biomedical Ethics Committees of Nankai University. The participant has given written informed consent to understand the study purpose, procedures, risks, benefits, and rights. The details of the SHIME system and the startup process are summarized in the Supplementary Material.

As shown in Supplementary Figure S1, during the first 2 weeks (0 to 14 days) of the experiment, control nutritional medium was added into the reactors to stabilize the microbial community. After this period, the SHIME was sequentially exposed to nutritious medium (15 to 21 days), and nutritious medium + 600 mg day^–1^ AMX (22 to 28 days), each time system was maintained for 1-week. Then a nutritious medium was added and observed for another 2-weeks (29 to 42 days). Samples were collected and analyzed at six time points of 14, 21, 24, 28, 35, and 42 days from the ascending colon, transverse colon and descending colon, respectively. Each of the 18 samples is a mixture of three samples collected at specific time intervals in a day (Liu et al., 2020), and AMX was added after samples (C-A2, C-T2, and C-D2) had been collected in 22 days and discontinued after samples (AMX-A2, AMX-T2, and AMX-D2) had been collected in 29 days. Therefore, these samples were divided into three groups: before AMX administration during 0 to 21 days (control group), AMX exposure during 22 to 28 days (AMX treatment group) and after the AMX discontinuance during 29 to 42 days (recovery group), which were according to previous classic studies (Yu et al., 2016; Wang et al., 2018). Specifically, during each sampling time, three sterilized centrifuge tubes (50 ml) were used to collect the samples (10 ml) flow out from each colon vessel of the SHIME, respectively, and these samples were initially stored at −4°C (Van de Wiele et al., 2010; Yu et al., 2016). After all the samples were collected in each sampling day, three samples from the same vessel were mixed into one sterilized centrifuge tube (50 ml), which was operated in a super-clean bench. Then the samples were centrifuged at 10,400 *g* for 10 min, and the separated supernatants and pellets were stored at −80°C for further analyses.

### 16S rRNA Gene Sequencing and Analysis

Total DNA was extracted from the samples using the E.Z.N.A. stool DNA Kit (Omega, United States) according to the manufacturer’s protocols. The V3–V4 region of the bacterial 16S rRNA gene was amplified by polymerase chain reaction (PCR) using primers 341F 5′-CCTAYGGGRBGCASCAG-3′ and 806R 5′-GGACTACNNGGGTATCTAAT-3′ (95°C for 2 min, followed by 27 cycles at 95°C for 30 s, 55°C for 30 s, and 72°C for 30 s, and a final extension at 72°C for 5 min). Amplicons were paired-end sequenced (PE250) on Illumina Hiseq2500 platform. In the end, a total of 651,502 tags were obtained. The raw reads were deposited into the NCBI Sequence Read Archive (SRA) database under the accession number of SRR9330193–SRR9330210. The details of bacterial DNA extraction and PCR amplification of the 16S rRNA gene are described in the Supplementary Material.

Raw Illumina fastq files were de-multiplexed, quality-filtered, and analyzed using Quantitative Insights Into Microbial Ecology (QIIME) (Caporaso et al., 2010). The 16S rRNA gene sequences were classified taxonomically using the Ribosomal Database Project (RDP), and classifier 2.0.1 (Wang et al., 2007). The AMX exposure on alpha diversity was further measured by the taxon richness (Chao1 index), evenness (Simpson index), and diversity index (Shannon index) using all recommended samples. Besides, beta diversity of microbiota communities at baseline and after antibiotics were portrayed by nonmetric multidimensional scaling (NMDS) and principal coordinate analysis (PCoA) of weighted and unweighted UniFrac distances. Linear discriminant analysis effect size (LEfSe) analysis was performed to determine the bacterial taxa that significantly differed between the control and AMX exposure group using Galaxy application tool (Segata et al., 2011). Functional predictions of microbial community were performed to visualize the distribution of functional pathway genes in the three parts of the colon with different treatments using Phylogenetic Investigation of Communities by Reconstruction of Unobserved States (PICRUSt) (Langille et al., 2013). The accuracy of PICRUSt for the detection of these more challenging functional groups was good (minimum accuracy = 0.82), suggesting that their inference of gene abundance across various types of functions was reliable, and PICRUSt predictions had high agreement with metagenome sample abundances across all body sites (Spearman *r* = 0.82, *P* < 0.001). PICRUSt has been successfully manipulated in many previous research for predicting microbial function of human intestinal microbiota (McHardy et al., 2013; Bunyavanich et al., 2016; Labus et al., 2017; Puri et al., 2018). The details of taxonomical classification, LEfSe analysis, and functional predictions are described in the Supplementary Material.

### High-Throughput Quantitative PCR (HT-qPCR) and Analysis

High-throughput quantitative PCR reactions were performed to visualize the variation of ARGs during the treatment using Wafergen SmartChip Real-time PCR system, conducted by Anhui MicroAnaly Gene Technologies Co., Ltd. (Anhui, China). A total of 108 primer sets were used (Excel S1), including 102 primer sets to target the almost all major classes of ARGs found in the Chinese human gut microbiota (Hu et al., 2013), five mobile genetic elements (MGEs), and one 16S rRNA gene. The results were analyzed with SmartChip qPCR Software by excluding the wells with multiple melting peaks or amplification efficiency beyond the range (90–110%). Then data were screened with the conditions that a threshold cycle (CT) must be <31 and positive samples should have three replicates simultaneously. The details of HT-qPCR analysis are described in the Supplementary Material.

### Data Analysis

All the results were expressed as mean values and standard deviations. The statistical analysis was performed with SPSS 17.0 software (SPSS Inc., Chicago, IL, United States). The *T*-test was conducted to compare the differences between the groups, and all the statistical tests were two-tailed. The statistical significance was set at three different levels (^∗^*P* < 0.05, ^∗∗^*P* < 0.01, and ^∗∗∗^*P* < 0.001). Spearman test, Mantel test, and Procrustes test for correlation analysis between the microbiota and the functional pathway genes or ARGs were performed in *R* with the vegan package. Correlations between the pairs of variables were considered to be significant at *R* > 0.6, and *P* values were <0.05. The Gephi (V 0.9.1) software was used to visualize the bipartite network graphs using the Force Atlas algorithm.

## Results

### AMX Exposure Increased Microbiota Diversity

**FIGURE 1:**
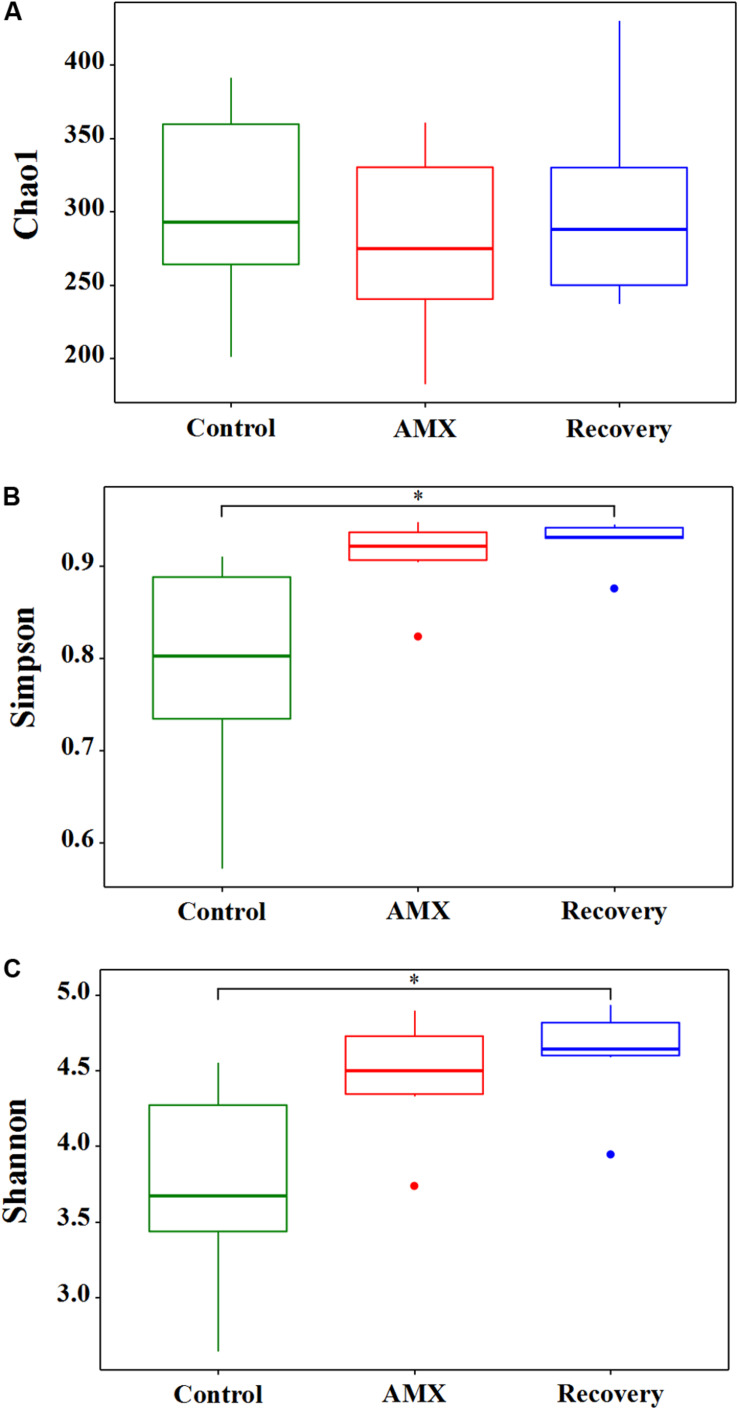
Effects of AMX treatment on gut microbiota alpha diversity of the control (green), AMX (red), and recovery (blue) groups. The Chao1 index **(A)** was used to calculate the community richness, Simpson index **(B)** was used to calculate the community evenness, and Shannon index **(C)** was used to calculate the community diversity within each of the three groups (control, AMX, and recovery). Statistical significance between each of the three groups were analyzed using the *T* test at a significance level of 0.05.

The effects of 600 mg day^–1^ AMX treatment on the gut bacterial community were revealed by the 16S rRNA gene sequencing of fecal samples collected from three different vessels designated as ascending, transverse, and descending colon. The vessels were set up for three groups: before AMX administration during 0 to 21 days (control group), AMX exposure during 22 to 28 days (AMX treatment group) and after the AMX discontinuance during 29 to 42 days (recovery group). The alpha diversity of the fecal microbiota was assessed in each group. The taxon richness (Chao1 index), evenness (Simpson index), and diversity index (Shannon index) of the three groups are shown in Figure 1. As compared with the control group, a substantially rising in the evenness (Simpson, *P* = 0.067, *T*-test) and diversity (Shannon, *P* = 0.061, *T*-test) was observed in the AMX treatment groups; however, no change occurred in the microbiota richness (Chao1, *P* = 0.564, *T*-test). Moreover, data displayed that an increasing of microbiota evenness and diversity caused by the AMX treatments was continued after two weeks of AMX discontinuance (Simpson, *P* = 0.044, *T*-test; Shannon, *P* = 0.028, *T*-test).

**FIGURE 2:**
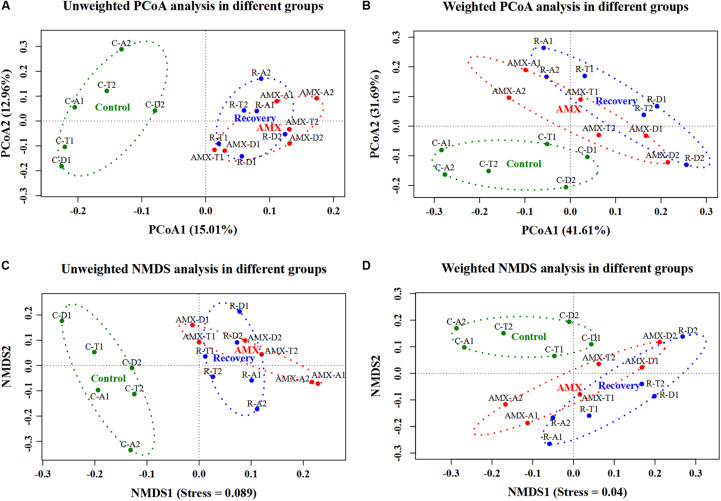
Effects of AMX treatment on gut microbiota beta diversity of the control (green), AMX (red), and recovery (blue) groups. Unweighted **(A)** and weighted **(B)** PCoA, and unweighted **(C)** and weighted **(D)** NMDS of UniFrac distances of samples in three groups (control, AMX, and recovery).

In addition, the beta diversity of the microbial communities and weighted UniFrac distance was also affected by AMX treatment. As shown in Figure 2 and Supplementary Figure S2, the samples collected after the AMX treatment were differed from the control group, because the two groups clustered far away from each other in both UniFrac NMDS and PCoA analyses. The average weighted UniFrac distance between the AMX treatment group and control group (AMX vs control) was significantly higher (*P* < 0.001, *T*-test) than that within the control group (control vs control). Similarly, the beta diversity results showed that the gut microbial composition remained comparable after 2-week of AMX discontinuance because these samples were clustered together with the AMX treatment groups observed in both UniFrac NMDS and PCoA analyses. The average weighted UniFrac distance between the recovery group and the control group (recovery vs control) was also significantly higher (*P* < 0.001, *T*-test) than the control group (control vs control).

### AMX Exposure Changed Microbiota Community Composition

**FIGURE 3:**
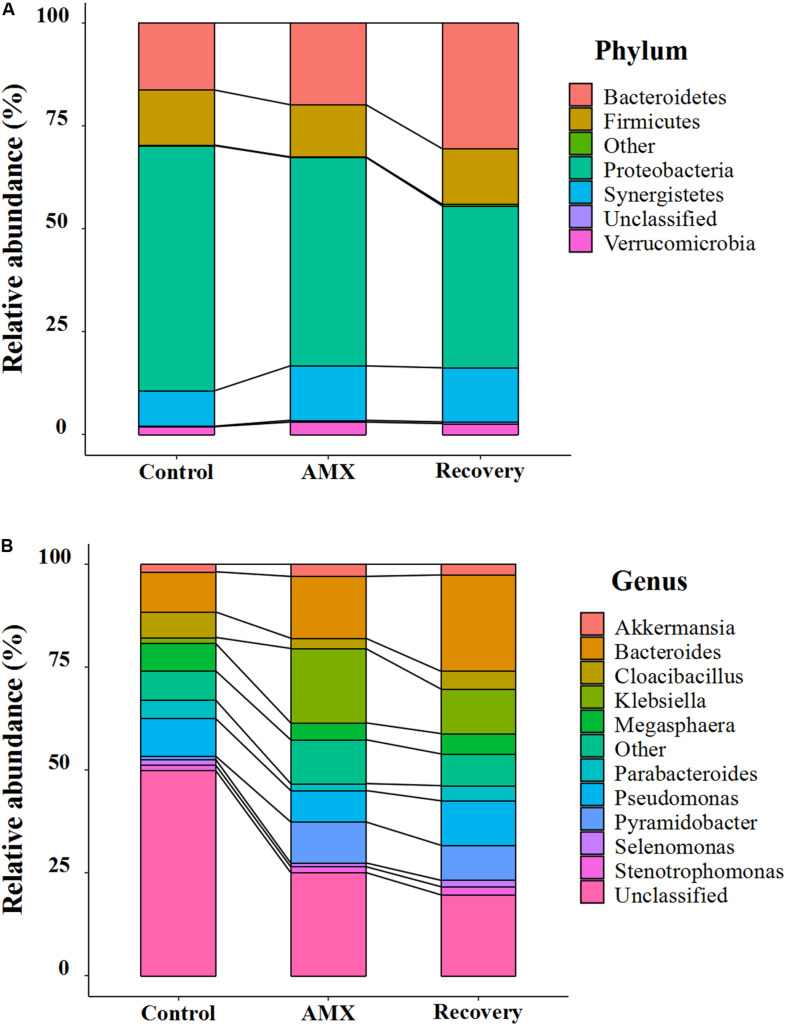
Composition shifts of gut microbial community at phylum **(A)** and genus level **(B)** between each of the three groups (control, AMX, and recovery).

The bacterial community compositions and their shifts at the phylum and genus levels after AMX exposure are shown in Figure 3 and Supplementary Figure S3. Based on the 16S rRNA gene analysis, the taxonomic assignment was related to most four dominant phyla such as *Bacteroidetes*, *Firmicutes, Proteobacteria*, and *Synergistetes*, which accounted for 96.5 to 97.9% of the total community (Figure 3A). However, a noticeable increase in abundance of *Bacteroidetes* (from 16.3 to 19.9%) and *Synergistetes* (from 8.6 to 13.2%), and decrease of *Proteobacteria* (from 59.6 to 50.7%) were seen after AMX treatment. The shifted phenomena (*Bacteroidete* from 13.3 to 23.2%; *Proteobacteria* from 68.8 to 57.8%) were more prominent in the ascending colon than the transverse and descending colons (Supplementary Figure S3A). A significant alteration in the communities was also observed at the phylum level (*Bacteroidetes* from 19.9 to 30.5%; *Proteobacteria* from 50.7 to 39.4%) after 2-week of AMX discontinued.

At the genus level, the *Bacteroides*, *Klebsiella*, *Megasphaera*, and *Pseudomonas* were predominant (Figure 3B). The antibiotic-treated subjects were shown to be substantially overgrown by *Bacteroides* (from 9.8 to 15.0%), *Klebsiella* (from 1.5 to 18.1%) and *Pyramidobacter* (from 0.8 to 10.0%), and declined in the percentage of *Cloacibacillus* (from 6.2 to 2.5%) and *Parabacteroides* (from 4.5 to 1.7%). Similarly, the changes of *Bacteroides* (from 8.4 to 20.5%) and *Klebsiella* (from 1.6 to 32.9%) were more evident in the ascending colon (Supplementary Figure S3B). As shown in Figure 3B, during the recovery period, it observed that the gut microbiota was not fully recuperated. Only, *Bacteroides* was inclined by abundance from 15.0 to 23.4%; however, all others were less retrieved (*Klebsiella* from 18.1% to 10.9%; *Pyramidobacte* from 10.0 to 8.5%; *Cloacibacillus* from 2.5 to 4.3%; *Parabacteroides* from 1.7 to 3.7%).

**FIGURE 4:**
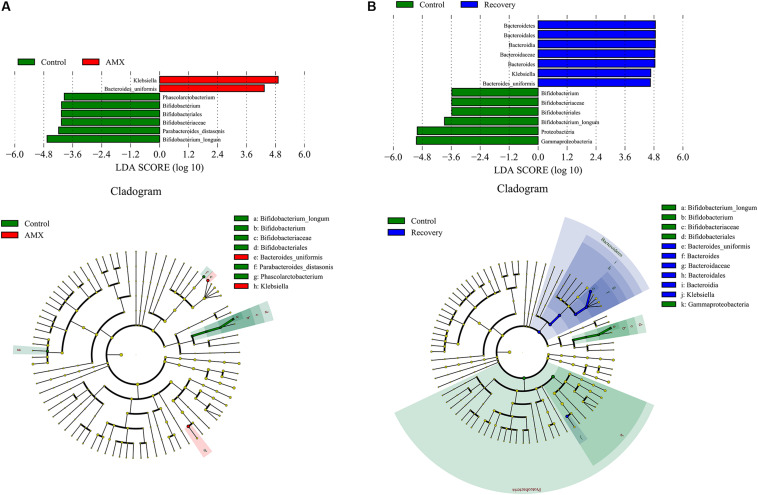
LDA score and cladogram of LEfSe comparison analysis between the control and AMX groups **(A)**, and between the control and recovery groups **(B)**. The red, green, or blue shading depicts bacterial taxa that were significantly higher in either the control, AMX or recovery groups, as indicated. Selection of discriminative taxa between the control and AMX groups or between the control and recovery groups were based on an LDA score cutoff of 3.0, and differences in the relative abundances of taxa were statistically determined based on a Mann–Whitney test at a significance level of 0.05.

The LEfSe showed that the AMX exposure caused an obvious decrease in several taxa, including the members of *Parabacteroides* [linear discriminant analysis (LDA) score = 4.18], *Bifidobacterium* (LDA score = 4.06), and *Phascolarctobacterium* (LDA score = 3.95) (Figure 4A). The variation was accompanied by significant increases in the relative abundances of *Klebsiella* (LDA score = 5.26), and *Bacteroides uniformis* (LDA score = 4.75). However, after 2-week of AMX discontinuance, the decrease of *Bifidobacterium* (LDA score = 3.88), and increase of *Klebsiella* (LDA score = 4.67) and *Bacteroides uniformis* (LDA score = 4.65) were still recognizable as compare with the control group (Figure 4B).

### AMX Exposure Increased Functional Pathway Genes

**FIGURE 5:**
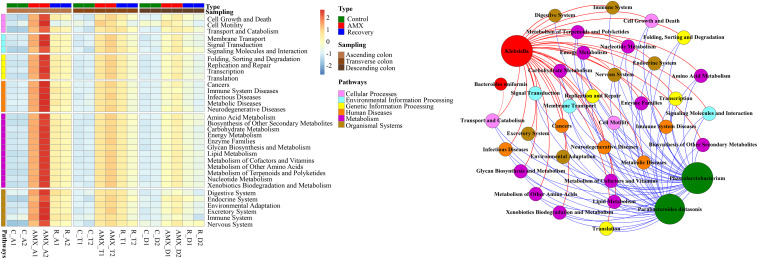
Heatmap of human disease-related pathways in the three parts of colon within three groups and the network analysis revealing the co-occurrence patterns between microbial taxa abundance and functional pathway gene number. Heatmap colors reflect relative abundance from low (blue) to high (red). The nodes in network were colored according to types of functional pathway genes and microbial genera that increased (red) or decreased (green) after AMX exposure, and the edges were colored according to positive (red) or negative (blue) correlation. A connection represents strong and significant (*P* value < 0.05, *R* > 0.6) correlation. The size of each node is proportional to the number of connections, that is, degree.

The hierarchy cluster heatmap analysis using metagenomic 16S rRNA gene sequencing was predicted by PICRUSt. Results showed that functional pathway genes, which included cellular processes, environmental information processing, genetic information processing, human diseases, metabolism, and organismal systems were more abundant in the antibiotic exposure group than in the control group that represented by the ascending colon (Figure 5). For instance, as compared to the AMX-free sample C-A2, collected from the ascending colon before AMX administration, the gene numbers of human diseases related-pathways were 2.4–3.8 times enriched in the AMX exposed sample AMX-A2 that obtained after AMX treatment for 7 days. Similarly, the numbers of genes respect to functional pathways, for examples, membrane transport, transcription, xenobiotics biodegradation, metabolism, and excretory system were nearly 4.6–5.3 folds enriched in the AMX-A2 sample than C-A2. Moreover, these pathways were still maintained at a higher level (approximately 2.2–2.5 times enhanced than the control group) after the recovery phase.

To investigate whether the OTUs correlated with the functional pathway genes, the Mantel test and Procrustes analysis were used. The results showed that OTUs from the ascending, transverse, and descending colons were moderately correlated with the numbers of functional pathway genes (Mantel test, *R* = 0.743, *P* < 0.001). The Procrustes analysis indicated that OTUs obtained from the 16S rRNA gene data and the functional genes could be clustered according to the type of sample, which further exhibited a goodness-of-fit test (*R* = 0.669, *P* < 0.001, and 9,999 permutations) based on the Bray–Curtis dissimilarity metrics (Supplementary Figure S4A). As shown in Figure 5, the results demonstrated the co-occurrence patterns of significantly shifted microbial taxa and functional pathway genes during AMX-treatment. It can be seen that the abundances of most imminent bacteria after AMX exposure such as *Bacteroides uniformis* and *Klebsiella* were positively associated with the gene numbers of functional pathways; however, the suppressed bacteria like *Phascolarctobacterium* and *Parabacteroides* were negatively associated (Figure 5). Specifically, the correlation coefficients of *Klebsiella* with the gene numbers of the digestive system, immune system, and metabolic diseases were about 0.98 (*P* < 0.001) and the correlation coefficients of *Phascolarctobacterium* abundance with gene numbers of signaling molecules and interaction, transport and catabolism, endocrine system, cell growth, and death were about −0.77 (*P* < 0.01).

### AMX Exposure Increased the Abundance of Beta-Lactam and Tetracycline Resistance Genes

**FIGURE 6:**
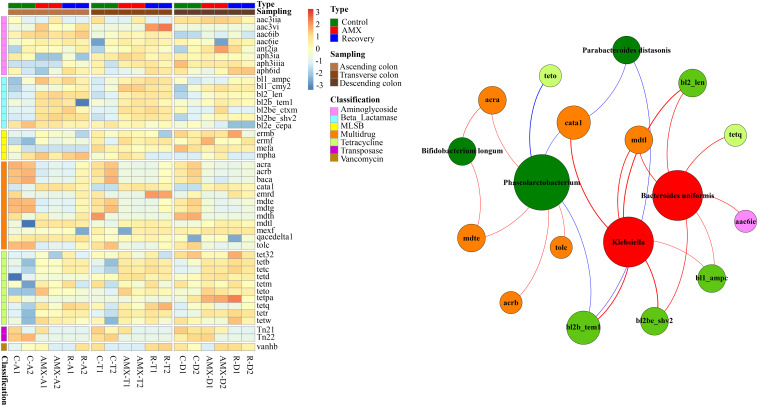
Heatmap of antibiotic resistance genes (ARGs) in the three parts of colon within three groups and the network analysis revealing the co-occurrence patterns between abundance of microbial taxa and that of ARG subtypes. Heatmap colors reflect relative abundance from low (blue) to high (red). The nodes in network were colored according to ARG types and microbial genera that increased (red) or decreased (green) after AMX exposure, and the edges were colored according to positive (red) or negative (blue) correlation. A connection represents strong and significant (*P* value < 0.05, *R* > 0.6) correlation. The size of each node is proportional to the number of connections, that is, degree.

The relative abundances of ARGs such as beta-lactam and tetracycline resistance genes were substantially higher in the AMX exposure group as compared to the control group, while the multidrug-resistant ARGs and transposase were lower in the AMX exposure group (Figure 6). Notably, the relative log abundance of *bl2_len, bl2b_tem1*, and *bl2be_ctxm* (aminoglycoside) were about 1.6–2.4 log units higher after AMX treatment (AMX-A2) than in control (C-A2). Similarly, the *tetb*, *tetc*, and *tetr* (tetracycline) were about 1.4–2.5 log units higher than the control group. Besides, the reductions of relative log abundance in the ARGs such as *mdte* and *tolc* (multidrug) were about 1.2 log units after AMX treatment (AMX-A2) than control (C-A2), and *Tn22* (transposase) was 3.0 log units. Similarly, these ARGs were unable to return at the baseline level following the recovery procedure with about 0.6 to 3.2 log units change of relative abundance compared with the control group.

To investigate whether the OTUs correlated with the resistome composition, Mantel test and Procrustes analysis were also performed to correlate the OTUs with the resistome using ascending, transverse and descending colons’ samples. Our results showed that OTUs were strongly correlated to the ARG profiles (Mantel test, *R* = 0.895, *P* < 0.001). The Procrustes analysis demonstrated that the bacterial OTUs and the ARGs in HT-qPCR data could be clustered by the type of sample and exhibited a goodness-of-fit-test (*R* = 0.941, *P* < 0.001, and 9,999 permutations) by Bray-Curtis dissimilarity metrics (Supplementary Figure S4B). The network analyses of co-occurrence patterns between the significantly changed microbial taxa after AMX treatment and ARG subtypes were shown in Figure 6. Interestingly, a very similar pattern of results was observed between the microbial taxa and functional pathway genes. The abundance of bacteria that significantly decreased after AMX treatment was positively associated with multidrug ARGs, while the abundance of increased bacteria was positively associated with beta-lactam resistance genes. For example, the moderate correlation coefficient of *Bifidobacterium longum* with *acra* abundance was 0.70 (*P* < 0.05), and for *Phascolarctobacterium* with *acrb* and *mdte* were 0.67 (*P* < 0.05). The strong correlations were found in the abundance of *Klebsiella* with the abundances of *bl2_len*, *bl2b_tem1*, and *bl2be_shv2* (*R* = 0.9, *P* < 0.001), and in *Bacteroides uniformis* with *bl2_len* and *bl2be_shv2* (*R* = 0.8, *P* < 0.01).

## Discussion

### AMX Treatment Has a Tremendous Influence on the Overall Taxonomic Composition and Biodiversity

The SHIME model was stably operated in this study because the predominant phyla of *Bacteriodetes*, *Proteobacteria*, *Synergistetes*, and *Firmicutes* in the gut microbiome was previously demonstrated Yu et al. (2016). *Firmicutes* and *Bacteroidetes* are usually dominate in the microbiota of a healthy subject, however in the control microbiota of SHIME, *Proteobacteria* is majoritarian. For *in vivo* studies, highest percentage of *Proteobacteria* had also been observed in fecal samples from healthy human and animals (Gao et al., 2015; Li et al., 2015). Our previous *in vitro* study also discovered this phenomenon (Liu et al., 2020).

It has been reported that *in vivo* experiments of AMX caused by tiny effects on human gut microbiota composition (Vrieze et al., 2014; Reijnders et al., 2016). However, a definite shift in the phylum level in our research may attribute to the absence of disturbances from neurohumoral regulation, the individual differences, dietary habits, and physiological status using *in vitro* SHIME (Karl et al., 2018). The decrease of *Proteobacteria* shown in our study may also attribute to AMX is more effective for sensitive Gram-negative bacteria that belonging to *Proteobacteria* by interfering with the synthesis of bacterial cell wall peptidoglycan layer (Zapata and Quagliarello, 2015). And *Bacteroides uniformis* belonging to *Bacteroidetes* may be resistant to AMX to make them survive, because of the strong correlations were found in their abundance with *bl2_len* and *bl2be_shv2* (*R* = 0.8, *P* < 0.01). Some studies even showed the opposite phenomena like increasing of *Proteobacteria* and decreasing of *Bacteroidetes*, which might be caused by the combined effects with other antibiotics such as clarithromycin, fosfomycin, and metronidazole (Oh et al., 2016; Ishikawa et al., 2017). However, at the genus level, the blooming of intestinal microbiota such as *Bacteroides uniformis* and *Klebsiella* and decrease of *Bifidobacterium* occurred following AMX administration. These data were further supported by earlier reports (Ladirat et al., 2014a; Oh et al., 2016; Marzorati et al., 2017), while an increase of *Parabacteroides* was found in another study (Kabbani et al., 2017).

Amoxicillin treatment affected a vast number of sensitive intestinal bacterial species that directly engrossed their corresponding functions. As a result, the functionally redundant resistant intestinal bacterial species were initially present at low levels and became more abundant. Thus, to compensate this loss of function to maintain community function, and species with closer evolutionary relationship usually have more similar functions (Moya and Ferrer, 2016). For example, Van den Abbeele et al. (2012) have found that during antibiotic treatment, the abundance of antibiotic-resistant *Pediococcus acidilactici* was enlarged, while the amount of sensitive *Lactobacillus mucosae* was declined. Similarly, in this study, a reduction of sensitive *Parabacteroides* might be replaced by resistant *Bacteroides* species. Although the metabolic function of the intestinal microbiota could recover quickly, the unrestored composition of the intestinal microbiota was supported by increasing ARB, which may pose a significant therapeutic challenge and that needs more attention.

As demonstrated by several studies that administration of antibiotics is significantly associated with decrease in microbial community diversity and richness, our results that AMX exposure increased the microbial diversity seems counterintuitive (Dethlefsen et al., 2008). The study by Vrieze et al. (2014) discovered a trend toward increased taxonomic diversity after AMX exposure, which was in accordance with our study. However, in some other studies no significant effect on microbiome diversity was found, which were opposed to our study (Pallav et al., 2014; Zaura et al., 2015; Reijnders et al., 2016). For this phenomenon, the possible reason was that AMX resistant intestinal bacterial species could bloom and compensate for or even surpassed the loss of AMX sensitive species. Intestinal microbiota with higher biodiversity was usually more resistant to the perturbation and colonization by pathogens, which might help in maintaining the intestinal homeostasis (Li et al., 2016). However, the improved biodiversity by AMX exposure that discovered in our study is mainly caused by increased abundance of AMX resistant intestinal bacterial species, which included opportunistic pathogen of the human intestine. This phenomenon may not be considered healthy for people because the increasing of opportunistic pathogens may be related to some human diseases that may cause the transmission and diffusion of ARGs.

### Pathogen Contribute to the Increased Human Disease Pathway Genes and Beta-Lactam Resistance Genes

Some research articles have not mentioned the significant alteration of metabolic functions of the gut microbiota after AMX treatment (Vrieze et al., 2014; Reijnders et al., 2016), while the disruption of the metabolic activity of microbiota (increased in succinate and monosaccharide and oligosaccharide levels) in the fecal samples were found elsewhere (Ladirat et al., 2014a). Moreover, this study found that *Klebsiella* was positively associated with the gene numbers of functional pathways including cancer, metabolic and immune diseases, and other human diseases, which have not been reported yet in other research articles. *K. pneumoniae* is a typical Gram-negative bacterial pathogen, which frequently colonizes in the human gut and processes to infection diseases (Pena et al., 1997; Wiener-Well et al., 2010; Gorrie et al., 2017). The increase of the relative abundance of intestinal *Klebsiella* genus has been reported to associate with diverse human diseases such as pneumonia, inflammation, Crohn’s disease, colitis, cystitis, liver abscess, and wound infections (Schneditz et al., 2014; Atarashi et al., 2017). All above findings suggested that *Klebsiella* might contribute to increase the pathway genes, including cancer, immune system diseases, infectious diseases, metabolic diseases, and neurodegenerative diseases.

It is also known from the literature that frequent use of antibiotics such as AMX may cause the transmission and proliferation of ARB and ARGs (Canton and Morosini, 2011; Zaura et al., 2015; Lenart-Boron et al., 2016). This study further demonstrated that the beta-lactamase resistance genes and the potential co-selected ARGs were increased after AMX exposure. The co-occurrence patterns between abundance of microbial taxa and that of ARG subtypes (Figure 6) showed that *Bacteroides uniformis* and *Klebsiella* might contributed to the increasing of ARGs including beta-lactam, tetracycline, and multidrug resistance. Therefore, treatment with AMX increases the number of resistance genes, even those not directly related to the antibiotic ingested. *Bacteroides uniformis* and *Klebsiella* are ubiquitous in the environment and are commensal in the human gut, notably the increased of their population, especially for *Klebsiella* is not usually considered as healthy. Moreover, resistance to beta-lactams via beta-lactamase production has mostly been described in *Klebsiella* spp. (Madhi et al., 2013; Marco et al., 2017). *Bifidobacterium* and *Parabacteroides* are most common or novel probiotics (Pinto-Sanchez et al., 2017; Wu et al., 2019), and the decreasing of these probiotics may cause dysbiosis of gut microbiota, which also leads to human health problem (Wischmeyer et al., 2016; George Kerry et al., 2018).

### AMX Caused “SHIME-Compartment” Different and Permanent Alterations

In this study, *Klebsiella* and *Bacteroides uniformis* were found to be more significantly enriched in the ascending colon than other two colonic-regions, which was in keeping with the previous reports of the alteration in intestinal microbiota by exposure of antibiotics mixture and other toxic compounds like chlorpyrifos and arsenic (Reygner et al., 2016; Yu et al., 2016; Marzorati et al., 2017). These studies revealed the “SHIME-compartment” specific effects that could be due to the inconsistent biodegradation of multiple compounds, gut microbiome community, and pH in different colon regions (Reygner et al., 2016; Yu et al., 2016; Marzorati et al., 2017). Moreover, a large number of functional pathways related genes was also shown to be increased in the ascending colon after AMX exposure, which further clarified that the primary effect observed at the level of the microbiota could also be identified at the genomic and metabolic levels (Garcia-Villalba et al., 2017; Wang et al., 2018). The intestinal microbiota is a key “organ” for the individual’s health, and its response to AMX is started from the proximal colon and hence observed more obvious shifts there. This phenomenon suggested that more studies should focus on the proximal colon; however, it may be challenging to study by *in vivo* experiments that usually analysis the feces standing for the distal intestinal microbiota.

Furthermore, these AMX effects were still evident for at least 2 weeks after the AMX discontinuance, although the resilient tendency of microbial composition, functional pathway genes and ARGs were also observed. Pallav et al. (2014) revealed that AMX mediated opportunistic pathogen such as *Escherichia/Shigella* persisted up to 42 days after the interruption of antibiotic therapy. Similar phenomenon was also reported by Zwittink et al. (2018), whose work primarily discovered a 5 days treatment with combined of AMX and ceftazidime allowed *Enterococcus* to thrive and remain dominant up to 2 weeks, while the abundance of *Bifidobacterium* remained decreased till postnatal of 6 weeks after antibiotic treatment discontinuation. Therefore, our results demonstrated a serious negative side-effect of AMX, which could be persistent and different in the specific colon region and should be considered as an essential aspect of the risk assessment for AMX prescription.

### PERSPECTIVES

Typical antibiotics have been detected in the collective gut of the Chinese population in our previous research, which provided a reference for this study of the effects of AMX on human gut microbiota (Wang et al., 2020). This study provides the originality of being able to control the effect of antibiotics in the different parts of the colon, evaluating in situ the alterations of the microbiota. Although SHIME has been successfully manipulated according to previous classic studies (Van den Abbeele et al., 2013; Yu et al., 2016; Wang et al., 2018), there were still three limitations of this study. First, AMX could not be absorbed by SHIME on the small intestine nor interact with the upper microbiota, which make the burden reaching the colon in the SHIME is much higher comparing to practical exposure to human colon. Considering that about 77 to 93% AMX is absorbed by the gastrointestinal tract and their interaction with the upper microbiota, the putative exposure dosage of AMX in colon is about 10% of the original oral dosage (Spyker et al., 1977; Arancibia et al., 1980; Huttner et al., 2019). This model is better to include the interaction with the human cell to get a closer result to *in vivo*, which is also a very good suggestion. Therefore, we would like to use 60 mg day^–1^ AMX (10% of this study) and include interaction with the human cell in our future work, with which the results would be close to *in vivo*. Second, this study lack of biological replicates and appropriate controls, which makes it difficult to interpret the results in relation to the microbial community variations and changes observed. Therefore, we would collect biological replicates and run a parallel experiment with blank medium at T0 and other timelines in our future study to negate any background noise and make the research scientifically reasonable. Another limitation is that the composition of the microbiota from the donor has not been analyzed before introducing it into the simulator. The composition of the initial microbiota from the donor may affect the stabilized microbiota in SHIME. Therefore, we would analyze the composition of the microbiota from the donor before introducing it into the simulator and compare the stabilized microbiota in SHIME that using multiple donors either by mixing their fecal samples or adding individually to explore the effects of initial microbiota composition in our future work, which would make this study more interesting. Although our work has these limitations, our findings would be valuable for directing future work. The findings in this study suggested several numbers of opportunities for additional study. One avenue is to expand the analysis to incorporate multi-omics approaches of the metagenome, metatranscriptome, and metabolome. Functional and metabolite analysis by multi-omics approaches would refine the results of predicting microbial function. Including interaction with the human cell in the fermentation vessels or manipulate *in vivo* study is also a good avenue to confirm the actual AMX’s effects on cancers, metabolic and immune diseases, and other human diseases that found in our *in vitro* experiments. On the other hand, the studies of virome and fungome may exert the substantial influences on the intestinal microbiome. Thus, an expanded analysis of other microorganisms will also be necessary. It is of interest to investigate the impacts of different kinds of antibiotics and a mixture of them on the gut microbiota. Moreover, research on the restoration effects of some prebiotics, probiotics, and synbiotics during and after antibiotic therapy is promising, which may help to discover several clinical strategies and restore side effects caused by antibiotics. As the increasing of opportunistic pathogens, functional genes and ARGs by antibiotics exposure may pose a significant therapeutic challenge, it should be more critical to take some efficient measures to reduce or even eliminate the effects caused by antibiotic treatment.

## Conclusion

Exposure to AMX had significantly altered the overall taxonomic composition of the gut microbiota with increasing taxonomic richness, functional pathway genes, and beta-lactam resistance genes. The changes were “SHIME-compartment” different and more substantial effect was observed in the ascending colon. The shifted human gut microbiota could not be restored after 2 weeks’ of AMX discontinued. Importantly, most of the functional pathway genes and quantified beta-lactam resistance genes are positively associated with bacteria that increased after AMX exposure. Our results may open up new perspectives for the assessing of direct effects by antibiotics on the intestinal microbiota – a key “organ” in individual health. The results demonstrated the negative side-effects of AMX and should be considered for AMX prescription.

## Data Availability Statement

The datasets generated for this study can be found in the NCBI SRA database, SRR9330193–SRR9330210.

## Ethics Statement

The study was approved by the Biomedical Ethics Committees of Nankai University. The participant has given written informed consent to confirm they understand the study purpose, procedures, risks, benefits, and rights.

## Author Contributions

LL carried out the laboratory work, analyzed the data, and wrote the manuscript. QW provided suggestions in the manuscript preparation and revised this manuscript. RD edited the language and improved the clarity of this manuscript. HL, SW, HQ, and JY carried out the laboratory work. YX provided funding support. DM and YL guided the laboratory work and revised this manuscript. All authors read and approved the final manuscript.

## Conflict of Interest

The authors declare that the research was conducted in the absence of any commercial or financial relationships that could be construed as a potential conflict of interest.
