# Supplementary material for: Amoxicillin Increased Functional Pathway Genes and Beta-Lactam Resistance Genes by Pathogens Bloomed in Intestinal Microbiota Using a Simulator of the Human Intestinal Microbial Ecosystem
Source: Front Microbiol. 2020 Jun 4;11:1213. doi: 10.3389/fmicb.2020.01213 (PMC7287123; doi:10.3389/fmicb.2020.01213)
Supplement: Supplementary file 2 [file Presentation_1.pdf]

Supplementary Material

Supplementary methods

SHIME model start up

All reactors were continuously stirred, maintained at 37 °C and kept under anaerobic conditions by flushing them with nitrogen for 30 min once a day (Figure S1). The pH values of the last two vessels of simulator were controlled in the ranges of 5.5 - 5.9, and 6.6 - 6.9, respectively. Three times a day, 200 mL of SHIME feed and 100 mL of pancreatic juice were added to the stomach and small intestine reactors, respectively. 1L of the SHIME feed contained 1 g arabinogalactan, 2 g pectin, 1 g xylan, 4 g starch, 0.4 g glucose, 3 g yeast extract, 3 g peptone, 1 g mucin, and 0.5 g cysteine, and 1L of pancreatic juice contained 12.5 g $NaHCO_3$ 6 g bile salts, and 0.9 g pancreatin (Yin et al., 2015).

DNA extraction

The DNA was eluted in a final volume of 50 µL using the E.Z.N.A. stool DNA Kit (Omega, USA). The quality of the DNA was checked by spectrophotometric analysis using NanoDrop (ND-2000, USA) and the concentration of DNA was determined using QuantiFluor® dsDNA system (Promega, USA) using fluorometric analysis with a microplate reader (Spectramax M5, USA). The extracted DNA was stored at −20 °C until use.

16s rRNA gene amplification

The V3-V4 region of the bacterial 16S rRNA gene were amplified by by polymerase chain reaction (PCR) (95 °C for 2 min, followed by 27 cycles at 95 °C for 30 s, 55 °C for 30 s, and 72 °C for 30 s and a final extension at 72 °C for 5 min) using primers 341F 5′- CCTAYGGGRBGCASCAG -3′ and 806R 5′-GGACTACNNGGGTATCTAAT -3′, where barcode is an eight-base sequence unique to each sample. PCR reactions were performed in triplicate with 20 µL reaction mixture contained 4 µL of 5 × FastPfu Buffer, 2 µL of 2.5 mM dNTPs, 0.8 µL of each primer (5 µM), 0.4 µL of FastPfu Polymerase, and 10 ng of template DNA.

Amplicons were gel run and extracted from 2% agarose gels. DNA was purified using the AxyPrep DNA Gel Extraction Kit (Axygen, USA) according to the manufacturer's instructions and quantified using QuantiFluor -ST (Promega, USA). The purified amplicons were pooled in equimolar and paired-end sequenced (2 × 250) on an Illumina platform according to the standard protocols.

16s rRNA sequence analysis

Sequences that were shorter than 55 bp, and contained primer mismatches, ambiguous bases or uncorrectable barcodes, were removed from the sequence data. The 16S rRNA gene sequences were assigned to operational taxonomic units (OTUs) using UCLUST with a threshold of 97% pairwise identity (Edgar, 2010), and then classified taxonomically using the Ribosomal Database Project (RDP) classifier 2.0.1 (Wang et al., 2007).

LEfSe analysis was performed using the Galaxy application tool (http://huttenhower.sph.harvard.edu/galaxy/) with a linear discriminant analysis, where the cutoff score is of 3.0 and a P of < 0.05 for statistical significance (Segata et al., 2011).

Functional predictions of microbial community were performed using Phylogenetic Investigation of Communities by Reconstruction of Unobserved States (PICRUSt) on closed-reference OTUs with 97% identity based on the Greengenes database (v13.5) (Langille et al., 2013). The OTUs were normalized on PICRUSt and used for the prediction of KEGG orthologs (KOs). These analyses were conducted by Guangzhou Gene Denovo Co., Ltd (Guangzhou, China).

High-throughput Quantitative PCR (HT-qPCR) and analysis

After the initial enzyme activation at 95 °C for 10 min, 40 cycles of the following program were used for the amplification: denaturation at 95 °C for 30 s, annealing at 60 °C for 30 s. The melting process was automatically generated by Wafergen software. After that, results were analyzed with SmartChip qPCR Software excluding the wells with multiple melting peaks or amplification efficiency beyond the range (90%–110%) and then screened with conditions that a threshold cycle (C_T) must be <31 and positive samples should have three replicates simultaneously.

Relative copy number was calculated referring to a previous study (Eq.1) (Ouyang et al., 2015). Besides, a comparative C_T method was used to calculate the ARGs' fold change (FC value) of amended samples compared to the control (Eq.2) (Schmittgen and Livak, 2008). The detection limit C_T (31) was taken as a replacement for the genes with no amplification.

$$Gene\ copy\ number = 10^{(31-C_T)/(10/3)} \qquad (1)$$

$$\Delta C_T = C_{T(ARG)} - C_{T(16S)}$$

$$\Delta\Delta C_T = C_{T(Target)} - \Delta C_{T(Ref)}$$

$$FC = 2^{-\Delta\Delta C_T} \qquad (2)$$

Where C_T is the threshold cycle, ARG is one of the 120 antibiotic resistance gene assays, 16S is the 16S rRNA gene assay, Target is the amended sample, Ref is the control sample.

Supplementary figures

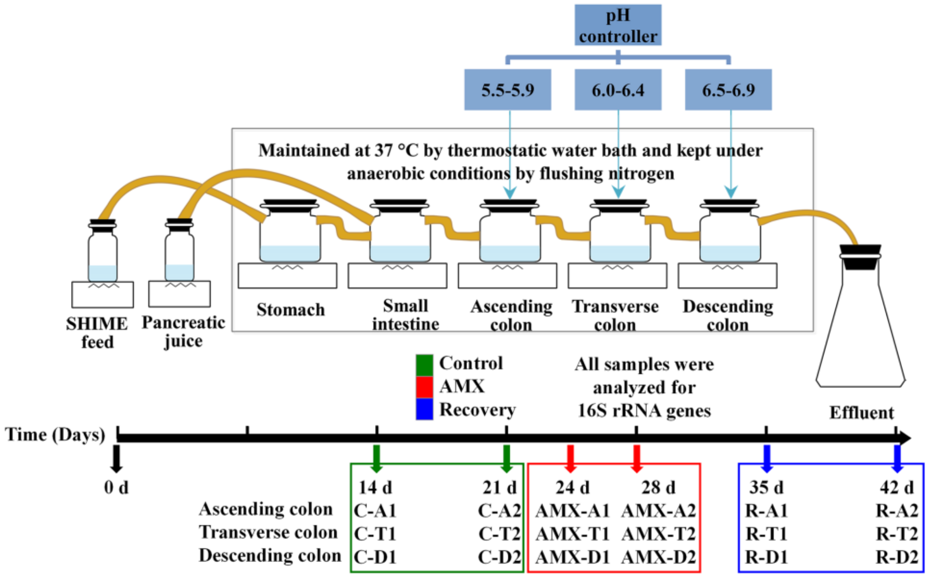

Supplementary Figure 1. Schematic of designed SHIME model and sampling time setup.

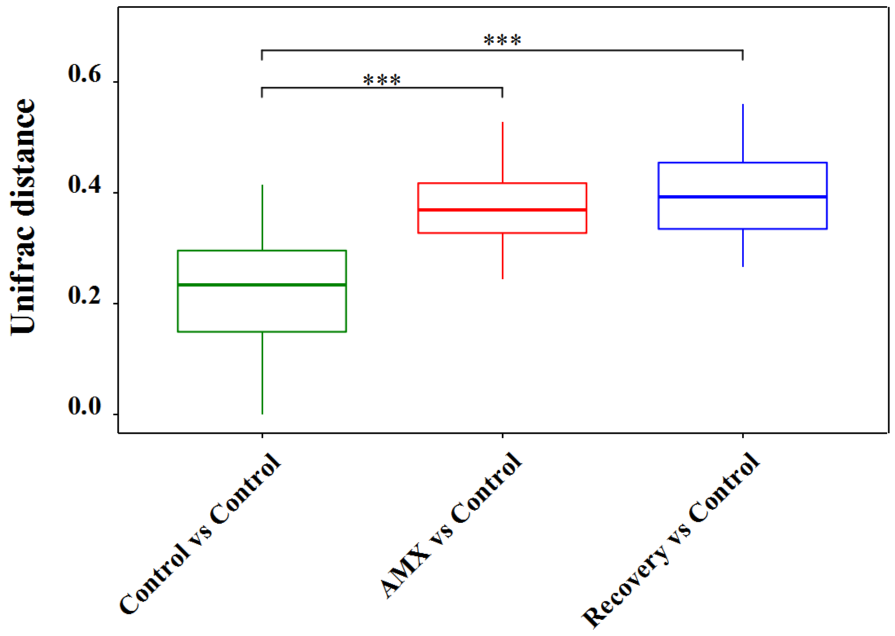

Supplementary Figure 2. Weighted UniFrac distance within control group (Control vs Control) and between treatment group and control group (AMX vs Control and Recovery vs Control). The *P* values were calculated using T test.

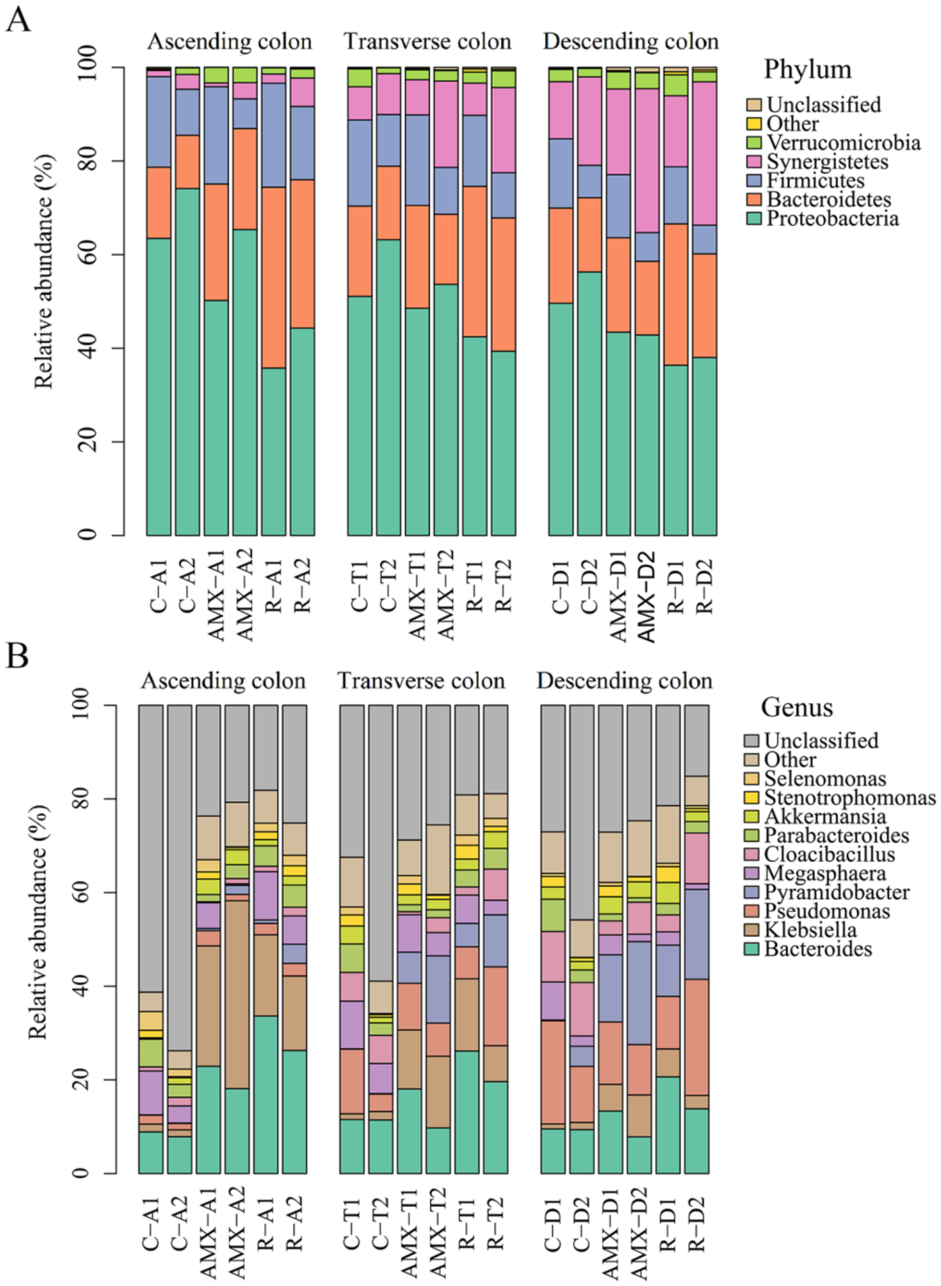

Supplementary Figure 3. Composition of microbial community at phylum (A) and genus level (B).

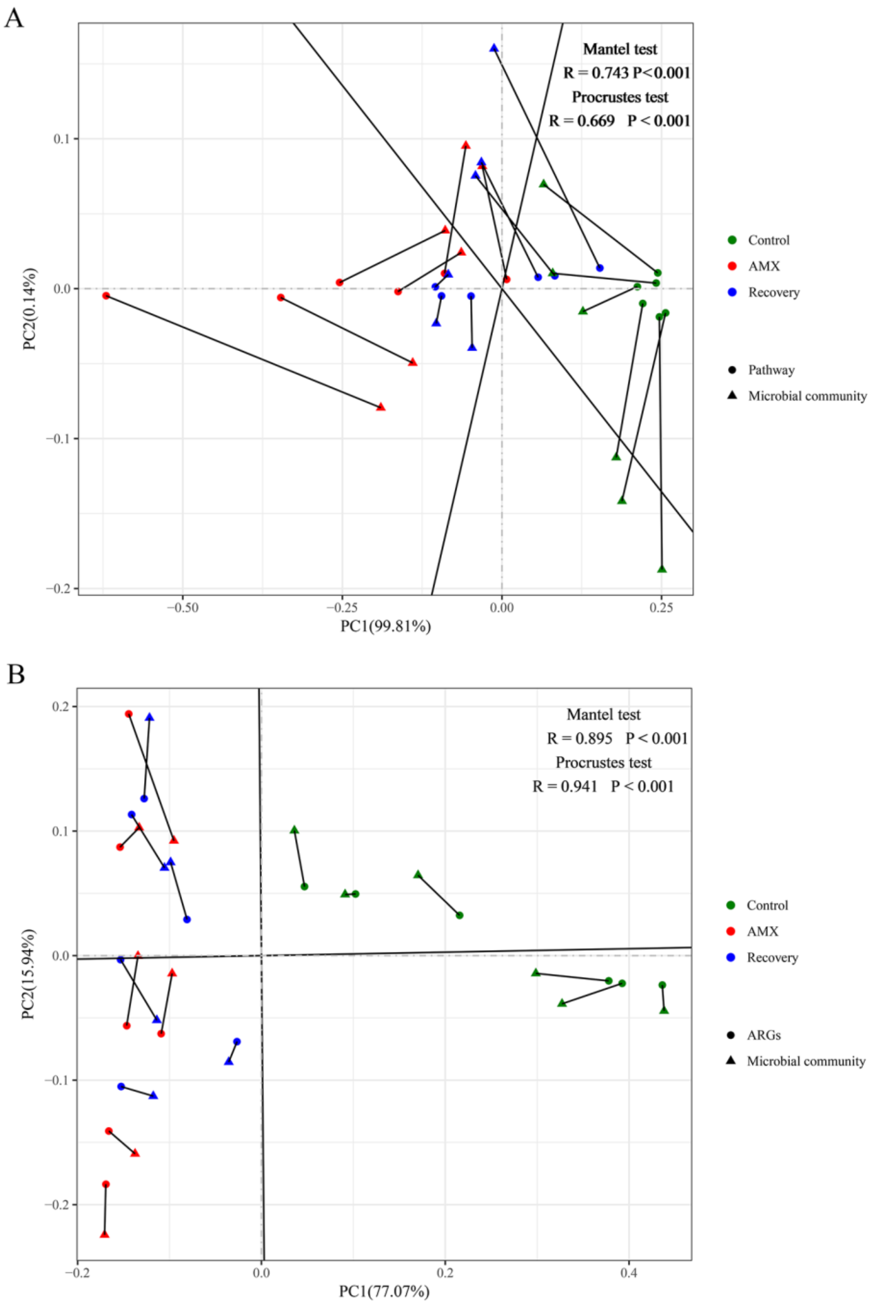

Supplementary Figure 4. Procrustes test depicting the significant correlation between bacterial taxa composition (16S rRNA gene OTUs data) and functional pathway genes (A) or ARGs (B) based on the Bray−Curtis dissimilarity metrics.

Supplementary References

Edgar, R.C. (2010). Search and clustering orders of magnitude faster than BLAST. *Bioinformatics* 26**,** 2460-2461. doi: 10.1093/bioinformatics/btq461.

Langille, M.G., Zaneveld, J., Caporaso, J.G., McDonald, D., Knights, D., Reyes, J.A., et al. (2013). Predictive functional profiling of microbial communities using 16S rRNA marker gene sequences. *Nat. Biotechnol.* 31**,** 814-821. doi: 10.1038/nbt.2676.

Ouyang, W.Y., Huang, F.Y., Zhao, Y., Li, H., and Su, J.Q. (2015). Increased levels of antibiotic resistance in urban stream of Jiulongjiang River, China. *Appl. Microbiol. Biotechnol.* 99**,** 5697-5707. doi: 10.1007/s00253-015-6416-5.

Schmittgen, T.D., and Livak, K.J. (2008). Analyzing real-time PCR data by the comparative C(T) method. *Nat. Protoc.* 3**,** 1101-1108. doi: 10.1038/nprot.2008.73.

Segata, N., Izard, J., Waldron, L., Gevers, D., Miropolsky, L., Garrett, W.S., et al. (2011). Metagenomic biomarker discovery and explanation. *Genome Biol.* 12**,** R60. doi: 10.1186/gb-2011-12-6-r60.

Wang, Q., Garrity, G.M., Tiedje, J.M., and Cole, J.R. (2007). Naive Bayesian classifier for rapid assignment of rRNA sequences into the new bacterial taxonomy. *Appl. Environ. Microbiol.* 73**,** 5261-5267. doi: 10.1128/Aem.00062-07.

Yin, N., Zhang, Z., Cai, X., Du, H., Sun, G., and Cui, Y. (2015). *In vitro* method to assess soil arsenic metabolism by human gut microbiota: Arsenic speciation and distribution. *Environ. Sci. Technol.* 49**,** 10675-10681. doi: 10.1021/acs.est.5b03046.
