# Supplementary material for: Amoxicillin Increased Functional Pathway Genes and Beta-Lactam Resistance Genes by Pathogens Bloomed in Intestinal Microbiota Using a Simulator of the Human Intestinal Microbial Ecosystem
Source: Front Microbiol. 2020 Jun 4;11:1213. doi: 10.3389/fmicb.2020.01213 (PMC7287123; doi:10.3389/fmicb.2020.01213)
Supplement: Supplementary file 3 [file Presentation_2.PDF]

医学伦理审查表

（Medical ethics approval）

申请日期(Date)：2016 年(Year)　9 月(Month)　13 日(Day)

项目名称(Project name)：应用 SHIME 系统研究抗生素对肠道菌群的影响<br>(An exploratory study of antibiotics effects on intestinal microbiota by SHIME)
项目负责人(Project leader)：罗义(Yi Luo)<br>职称(Job titles)：教授(Professor)<br>单位(Institution)：南开大学(Nankai University)
项目联系人(Project contact)：罗义(Yi Luo)　　电话(Tel.)：86-22-85358553<br>信箱(E-mail)：luoy@nankai.edu.cn
请求审查类型(Review type)：<br>☑申请项目(Application for project)　☐批准后项目(Approved project)<br>☐延续项目(Continuation of the project)　☐委托项目(Entrusted project)
研究项目来源(Fund for research)：<br>国家自然科学基金(National Natural Science Foundation of China)
**涉及人的生物医学研究内容及研究方案摘要(Biomedical research involving people and abstract of research content)**<br>本项目采集中国健康人群（6 个月内未感染胃肠疾病且 6 个月内未服用过抗生素）的粪便样本，接种到人体胃肠道生态系统模拟器（SHIME），运行系统研究抗生素处理对肠道菌群的影响。本项目研究成果能有助于对肠道菌群受抗生素影响的认知，以及对临床合理利用抗生素提供帮助指导。<br>(We collected fecal samples from healthy individuals in China (who did not suffer from gastrointestinal diseases or take antibiotics in the previous six months) and inoculated fecal samples into SHIME to study the antibiotics effects on intestinal microbiota. This research could contribute to the cognition of antibiotics effects on intestinal microbiota and implicate a promising approach in guidance to clinical antibiotic prescription.)

医学伦理审查意见(Medical ethics review comments)

经审查，该研究项目符合卫生部《涉及人的生物医学研究伦理审查办法（试行）》及赫尔辛基宣言关于生物学人体试验的相关规定，同意开展研究。

(This research complied with the ethical review of biomedical research involving human (trial) of Ministry of Health in China. It has been performed in accordance with the Declaration of Helsinki and has been approved by local ethics committee.)

开大

单位公章(Official seal)

2016 **年(Year)** 9 **月(Month)** 13 **日(Day)**
