# Supplementary material for: Amoxicillin Increased Functional Pathway Genes and Beta-Lactam Resistance Genes by Pathogens Bloomed in Intestinal Microbiota Using a Simulator of the Human Intestinal Microbial Ecosystem
Source: Front Microbiol. 2020 Jun 4;11:1213. doi: 10.3389/fmicb.2020.01213 (PMC7287123; doi:10.3389/fmicb.2020.01213)
Supplement: Supplementary file 4 [file Presentation_3.PDF]

参与者知情同意书

The informed consent of participants

尊敬的参与者：

经调研，您是《应用 SHIME 系统研究抗生素对肠道菌群的影响》的合格纳入者，并被邀请参加本项目研究。您可自愿参加研究，签署知情同意书。在项目任何阶段，您都有权利随时离开项目。您需要提供真实的问卷信息，所有信息（个人信息，检查结果等），我们都会为您保密，不会泄露给第三方。本研究样品来自于人体肠道粪便样本剩余，对您不会有任何额外风险。

Respectable participants:

You are a qualified participant in the study of "An exploratory study of antibiotics effects on intestinal microbiota by SHIME". We invite you for this study and sign the informed consent. You are entitled to leave this study at any time. You need to provide questionnaire and it will never be available to third party. In this research, samples are from the remaining fecal samples and there is not any additional risk to you.

工作人员声明：我已经向调查对象宣传和解释了这份知情同意书，他/她 已经理解并同意参加本项目。

The staff statement: I have explained the informed consent to respondents, and he/she has understood and agreed to participate in the study.

工作人员签字(Signature of the staff statement) [illegible]

(Date) 2016 年(Year) 9 月(Month) 21 日(Day)

自我声明：我已经阅读本知情同意书，理解全部相关情况，一些问题已经同项目工作人员进行讨论并得到满意答复，我同意参加本次研究。

Self-declaration: I have read the informed consent and understand all the content. Some issues have been discussed with the project staff and get a satisfactory answer. I agree to participate this study.

[illegible]

参与者签字(Signature of participant)

(Date) 2016 年(Year) 9 月(Month) 21 日(Day)
